# Toxicity Characterisation of *Gambierdiscus* Species from the Canary Islands

**DOI:** 10.3390/toxins12020134

**Published:** 2020-02-21

**Authors:** Araceli E. Rossignoli, Angels Tudó, Isabel Bravo, Patricio A. Díaz, Jorge Diogène, Pilar Riobó

**Affiliations:** 1 Instituto Español de Oceanografía, Centro Ocenográfico de Vigo, Subida a Radiofaro 50, 36390 Vigo, Spain; isabel.bravo@ieo.es; 2 IRTA, Ctra. Poble Nou, km. 5.5, 43540 Sant Carles de la Ràpita, Spain; angels.tudo@irta.cat (A.T.); jorge.diogene@irta.cat (J.D.); 3 Centro i~mar & CeBiB, Universidad de Los Lagos, Casilla 557, Puerto Montt, Chile; patricio.diaz@ulagos.cl; 4 Department of Photobiology and Toxinology of Phytoplankton, Instituto de Investigaciones Marinas, CSIC, Eduardo Cabello 6, 36208 Vigo, Spain; pilarriobo@iim.csic.es

**Keywords:** Canary Islands, ciguatera, ciguatoxins, erythrocyte lysis assay, maitotoxins, neuroblastoma cytotoxicity assay

## Abstract

In the last decade, several outbreaks of ciguatera fish poisoning (CFP) have been reported in the Canary Islands (central northeast Atlantic Ocean), confirming ciguatera as an emerging alimentary risk in this region. Five *Gambierdiscus* species, *G. australes*, *G. excentricus*, *G. silvae*, *G. carolinianus* and *G. caribaeus*, have been detected in macrophytes from this area and are known to produce the ciguatoxins (CTXs) that cause CFP. A characterization of the toxicity of these species is the first step in identifying locations in the Canary Islands at risk of CFP. Therefore, in this study the toxicity of 63 strains of these five *Gambierdiscus* species were analysed using the erythrocyte lysis assay to evaluate their maitotoxin (MTX) content. In addition, 20 of the strains were also analysed in a neuroblastoma Neuro-2a (N2a) cytotoxicity assay to determine their CTX-like toxicity. The results allowed the different species to be grouped according to their ratios of CTX-like and MTX-like toxicity. MTX-like toxicity was especially high in *G. excentricus* and *G. australes* but much lower in the other species and lowest in *G. silvae*. CTX-like toxicity was highest in *G. excentricus*, which produced the toxin in amounts ranging between 128.2 ± 25.68 and 510.6 ± 134.2 fg CTX1B equivalents (eq) cell^−1^ (mean ± SD). In the other species, CTX concentrations were as follows: *G. carolinianus* (100.84 ± 18.05 fg CTX1B eq cell^−1^), *G. australes* (31.1 ± 0.56 to 107.16 ± 21.88 fg CTX1B eq cell^−1^), *G. silvae* (12.19 ± 0.62 to 76.79 ± 4.97 fg CTX1B eq cell^−1^) and *G. caribaeus (*<LOD to 90.37 ± 15.89 fg CTX1B eq cell^−1^). Unlike the similar CTX-like toxicity of *G. australes* and *G. silvae* strains from different locations, *G. excentricus* and *G. caribaeus* differed considerably according to the origin of the strain. These differences emphasise the importance of species identification to assess the regional risk of CFP.

## 1. Introduction

*Gambierdiscus* is a genus of marine benthic dinoflagellates that produces maitotoxins (MTXs) and ciguatoxins (CTXs) [1,2,3]. These cyclic polyether neurotoxins are among the five most potent natural toxins isolated to date [4]. Because they accumulate in coral reef fish, they can be transferred through the marine food web [5,6,7,8]. In fact, CTXs are the main toxins responsible for ciguatera fish poisoning (CFP) [9], a clinical syndrome caused by eating CTX-contaminated fish. The risks to human health related to the consumption of these toxins in fish have been assessed by the EU Food Safety Authority (EFSA) Panel on Contaminants [10]. CFP is widespread in tropical and sub-tropical marine areas, including the Caribbean Sea, Indian Ocean, Polynesia and other areas of the Pacific Ocean [11]. However, over the past decade, an increase in the incidence of CFP in areas where ciguatera is endemic [12,13,14] and several outbreaks of ciguatera in more temperate regions, such as Madeira [15,16,17] and the Canary Islands (NE Atlantic Ocean) [18], suggest an expansion of this disease is occurring.

The first reported CFP outbreak in the Canary Archipelago was described in 2004. Five people were affected [19]. Two additional episodes happened in 2008 causing the intoxication of 11 people [20]. Since then, several CFP episodes affected 113 people (Canary Government, 2017), [21,22]. Because of recurrent outbreaks, CFP has been designated as a notifiable disease in the Canary Islands since 2015 (Canary Government, 2017).

The main fish species involved in CFP in this area are amberjack (*Seriola spp*.), dusky grouper (*Epinephelus marginatus*) and Wahoo (*Acantocybium solandri*). The weight limits above which risks might occur are 14 kg, 17 kg and 35 kg respectively. Although CFP occurs when toxic fish is ingested, CTXs are produced by microalgae belonging to *Gambierdiscus* genus.

In the Canary Islands, ciguatera outbreaks have been related to *Gambierdiscus* species, including *G. australes*, *G. excentricus*, *G. silvae*, *G. carolinianus* and *G. caribaeus* [23], all of which have been isolated from macrophytes. An expansion of the *Gambierdiscus* distribution toward higher latitudes has been attributed to the increase in the ocean temperature caused by climate change.

CTXs are selective activators of voltage-dependent Na^+^ channels in cells [24,25,26,27] whereas MTXs are water-soluble and alter the ion transport systems, causing an increase in free intracellular Ca^2+^ [28,29,30,31,32]. Although highly toxic [33], MTXs do not induce CFP, because of their low oral potency and inability to accumulate in the muscle tissue of fish [7]. The extensive literature survey by Munday [34] found no published records of the oral toxicity of MTX and the only evidence of MTX accumulation in fish liver and viscera recorded before 2014 came from two studies published in the 1970s [35,36]. Whether MTX can cause CFP via other routes remains to be investigated.

Numerous methodologies based on different approaches (e.g., toxicological symptoms, antibody recognition, mass spectrometry, etc.,) have been developed for the detection of CTX and MTX, but their use is often problematic (due among other things to, antibody cross-reactivity for CTX from different origins). In addition to better sampling procedures to allow an efficient extraction and concentration of the toxins, improved clean-up procedures to remove impurities that negatively impact sample analyses are needed. From an analytical perspective, the complexity of the sample matrix, the very low levels of toxin detected, and the lack of reference toxin material have greatly hampered the development of reliable methods for CTX and MTX determinations. Moreover, there are currently no chemical methods with the required sensitivity and specificity to rapidly monitor either of these toxins [37], and detection by LC-MS/MS [38] may lead to misidentifications. Consequently, to date, CTXs have been identified only in a few *Gambierdiscus* strains, i.e., those of *G. toxicus* [39], *G. polynesiensis* [2], *G. ruetzeri* [40], *G. pacificus* [3], *G. silvae* and *G. excentricus* [41]. In the absence of a rapid, cost-effective and reliable screening test for CTXs, health authorities around the world have relied on guidelines aimed at preventing high-risk fish species and fish life stages from entering the commercial market to reduce the risk of CFP [42]. This strategy takes into account that CTXs can bioaccumulate over time, such that older and larger fish are more likely to be contaminated with higher levels of CTXs. However, few studies have directly examined the relationship between fish size and the presence of CTX in individual fish species [43]. A further problem is that there are no accredited methods for the analysis of CTXs and MTXs in fish samples.

**Figure 1:**
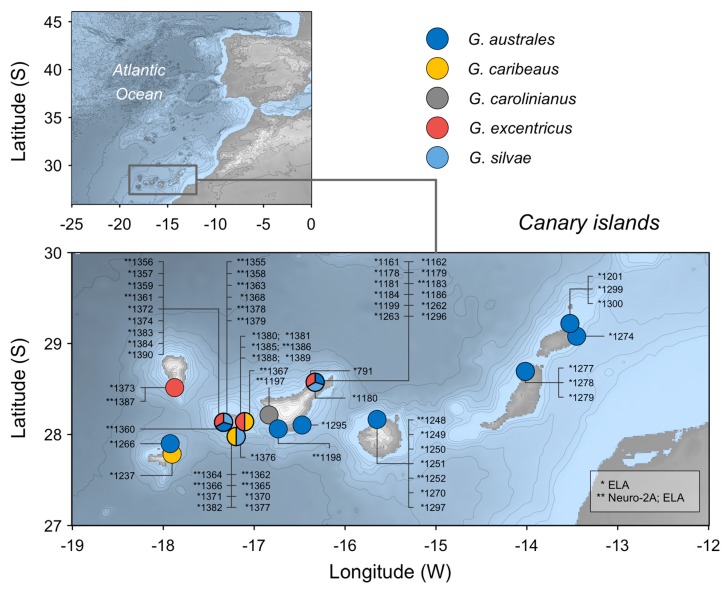
*Gambierdiscus* strains from Canary Islands examined in this study. * Analysed using the erythrocyte lysis assay (ELA); ** analysed using both the ELA and the Neuro-2a cell assay.

A previous study showed that toxicity is more closely related to genetics rather than to environmental (temperature, ph and salinity) factors [44]. Among *Gambierdiscus* species in the Canary Islands, both *G. excentricus* [45,46] and *G. australes* [47] are known to produce high levels of CTX. However, information on other regional members of this genus is scarce. Therefore, in this study we characterised the relative toxicity of 63 *Gambierdiscus* strains of the five *Gambierdiscus* species described in the Canary Islands thus far (Figure 1). An erythrocyte lysis assay (ELA) based on the method of Holland et al. [48] was performed to determine and compare MTX production by the tested strains. In addition, CTX production by the 20 most representative strains was assessed using the neuroblastoma cell-based assay (Neuro-2a assay). Compared to instrument-based methods, the ELA and the Neuro-2a assay are more sensitive and do not require precise standards, since both assess the effect of the toxins on cells. Consequently, the assays have been widely used to detect MTX-like and CTX toxicities, respectively. The particular advantages of the ELA as a screening assay is that it requires relatively little starting material and is fast, highly reproducible and cost-effective.

## 2. Results

### 2.1. Screening of CTX-Like Toxicity Using the Neuro-2a Assay

**Figure 2:**
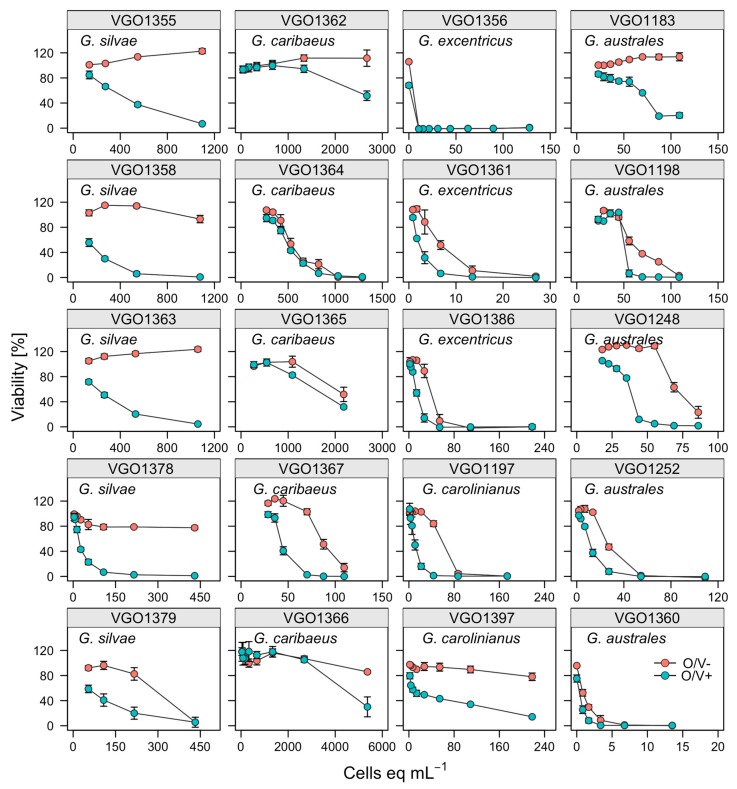
Dose–response curves of treated (O/V + green symbol) and non-treated (O/V−, red symbol) neuroblastoma cells 24 h after their exposure to extracts prepared from 20 *Gambierdiscus* strains.

As predicted, when neuroblastoma cells were exposed to CTX1B standard they did not exhibit toxicity signals (>80%) in O/V− conditions. Conversely when neuroblastoma cells were exposed to CTX1B in O/V+ treatment, the cell viability exhibited a typical sigmoid dose-response curve. The IC_50_ mean for the CTX1B standard was 2.33 ± 1.30 pg CTX1B mL^−1^. The crude extracts (CE) of nineteen canaries strains (Figure 1) and the Cuba strain VGO1397 were tested with the neuro-2a assay. CTX-like toxicity was attributed to microalgal extracts when the addition of a sample extract to neuroblastoma cells exposed to O/V+ treatment resulted in 20–80% viability decrease. However, the addition of the same extract to neuroblastoma cells exposed to O/V− treatment did not affect the cell viability (>80% viability). Moreover, enhanced mortality in the wells in O/V− conditions were observed (<80% of viability) for some strains. This indicates the presence of non-specific toxic compounds other than sodium channel activators that apparently is according to the results of the ELA analysis. These non-specific effects are shown in Figure 2 showing the dose-response curve of 20 Gambierdiscus strains tested in the Neuro-2a cellular assay.

Neuro-2a cells, exposed to *G. excentricus* extracts (VGO1356, VGO1361, VGO1386) at concentrations ranging between 10.5 and 54 microalgal cells eq mL^−1^ with O/V−, showed a very low viability (<20%). The activity of *G. australes* extracts presented higher variability in toxicity responses as compared to *G. excentricus*. Four extracts (VGO1198, VGO1248, VGO1252, VGO1360), at concentrations ranging between 80 and 100 cells mL^−1^ in O/V−conditions, affected the cell viability highly. However extract (VGO1183) at the same concentrations and conditions did not affect the cell viability. This variability in O/V− conditions could be observed also in *G. caribaeus* and *G. silvae* extracts. For *G. caribaeus*, 3 out of 5 strains affected Neuro-2a viability at levels ranging from 70 to 1000 cells eq mL^−1^ (VGO1364, VGO1365, VGO1367). For *G. silvae*, 1 out of 4 strains (VGO1379) affected Neuro-2a viability at approximately 500 cells eq mL^−1^ (Figure 2). For the other strains of *G. caribaeus* (VGO1366) and *G. silvae* (VGO1358), no signals of toxicity under O/V− conditions appeared at 3000 cells eq mL^−1^ and 1000 cells eq mL^−1^, respectively (Figure 2).

**Figure 3:**
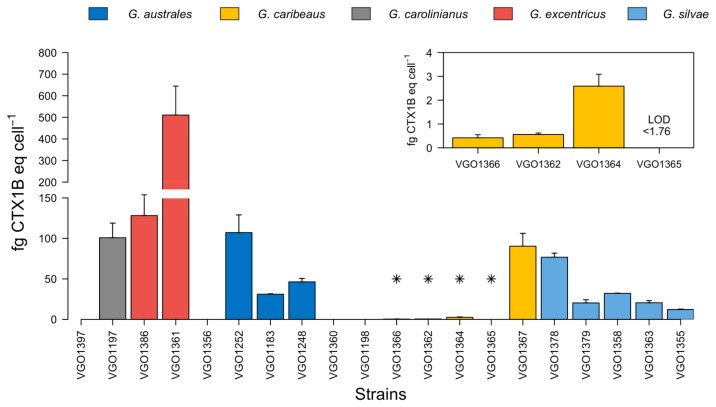
CTX-like toxicity expressed in femtograms of CTX1B equivalents per cells (fg CTX1B eq cell^−1^) of *Gambierdiscus* strains as determined in a Neuro-2a cytotoxicity assay. Strains with the lowest concentrations are indicated by asterisks (*) and shown in the small bar plot in the inset. Strains VGO1397 (*G. carolininanus*), VGO1356 (*G. excentricus*), VGO1360 and VGO1198 (*G. australes*) could not be quantified. Bars in the columns represent the standard deviation.

A quantitative estimation of CTX-like toxicity was possible in only 16 of the 20 sample analysed, despite the very low cell viabilities (<20%) in the O/V− treatments exposed to VGO1198 and VGO1360 (*G. australes*), VGO1356 (*G. excentricus*) and VGO1397 (*G. carolininanus*), the cell viability plots did not result in a sigmoidal curve (Figure 2). The results for the other strains are expressed as fg CTX1B eq cell^−1^ (Figure 3). Among these strains, the highest levels of CTX-like toxicity (between 128.2 ± 25.68 and 510.6 ± 134.2 fg CTX1B eq cell^−1^) occurred in response to *G. excentricus* extracts, followed by VGO1197 *G. carolinianus* extracts (100 ± 18.05 fg CTX1B eq cell^−1^) and *G. australes* and *G. silvae* extracts (from 31.1 ± 0.56 to 107.16 ± 21.88 and from 12.19 ± 0.62 to 76.79 ± 4.97 fg CTX1B eq cell^−1^, respectively) (Figure 3). The lowest toxicity occurred in response to extracts from the *G. caribaeus* strains (from below the detection limit (1.76) to 2.59 ± 0.5 fg CTX1B cell^−1^ eq) (Figure 3). However, *G. caribaeus* VGO1367, the only strain whose origin (La Gomera, San Sebastián-Playa la Cueva) differed from that of other strains of the same species (La Gomera Porto-Playa Santiago), had a high CTX-like toxicity (90.37 ± 15.89 fg CTX1B eq cell^−1^) (Figure 3).

### 2.2. Toxicity Screening Using the Erythrocyte Lysis Assay

**Figure 4:**
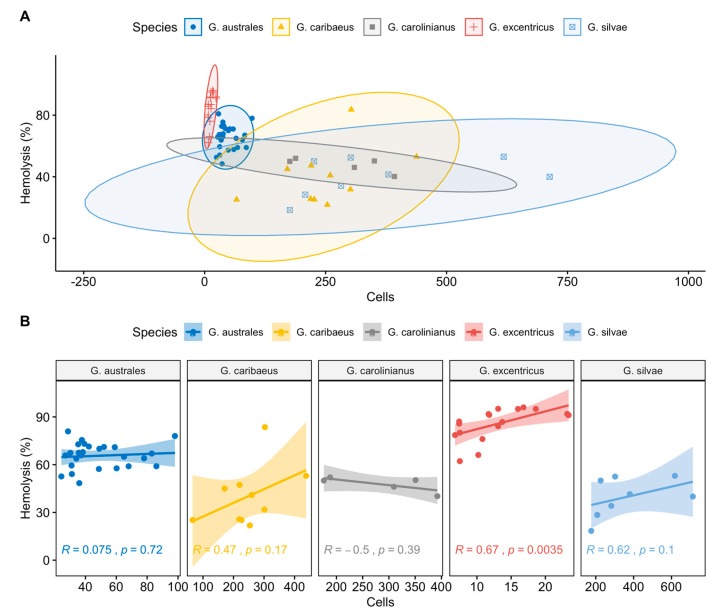
Scatter plot (**A**) and line regression (**B**) of the relationship between cell abundance and haemolysis (%) for each *Gambierdiscus* strain. Ellipses and shaded areas in panels A and B represent the 95% confidence intervals. Correlation coefficients (*R*) and *p*-values are indicated in each line regression plot.

The relationship between cell abundance and haemolysis (%) for each strain of the five *Gambierdiscus* species (*G. australes*, *G. caribaeus*, *G. carolinianus*, *G. excentricus* and *G. silvae*) is shown in Figure 4A,B. Among the tested strains of *G. excentricus*, cell abundance correlated significantly with haemolysis (*p* < 0.05; R = 0.67, Figure 4B). The analysis of covariance (ANCOVA) showed the significant effects of cell abundance and *Gambierdiscus* species on the percent haemolysis (Table 1). There was no interaction between two explicative variables.

Post-hoc Tukey’s H comparisons of the percent haemolysis induced by the five *Gambierdiscus* species showed significant differences, with higher MTX like toxicities associated with *G. excentricus* and *G. australes* than with *G. caribaeus*, *G. carolinianus* and *G. silvae* (Table 2).

## 3. Discussion

The Canary Islands are a recent area of ciguatera endemicity [18]. Benthic dinoflagellates belonging to the *Gambierdiscus* genus, specifically those that could produce MTXs and CTXs, seem to be implicated in ciguatera outbreaks in the region and therefore the risk of seafood poisoning. Vulnerable fish species include *Seriola* spp. and *Epinephelus* spp.

Tools to assess the ciguatera risk in different areas based on the toxins detection should be fast and have a low detection limit. In addition they should be able to detect the different toxins analogues and derivatives. Neuroblastoma cell-based assay accomplishes very well the last two requirements. Neuroblastoma cell-based assay can detect the effect of CTXs and MTXs analogues at low concentrations [49,50,51,52] and also other possible novel toxins with the same effect on sodium channels [53]. The drawback of this is that it cannot attribute the CTX-like or MXT-like effect to one specific molecule [52]. For this, instrumental techniques such as LC-MS/MS or LC-HRMS are required. In the present study, the cell-based assay was applied as a first screening tool to be used for the higher risk species through the comparison of the strains composite CTX-activity and MTX-activity.

Two approaches to evaluate the toxicity of the extracts in neuroblastoma cells were used in this study. The first one is neuroblastoma cell viability evaluation in absence of ouabain and veratridine. Under this approach, cell death may not indicate the presence of a specific type of compound. This is called non-specific toxicity. MTXs production has been described in *Gambierdiscus* species [46,54,55]. The non-specific toxicity in the absence of ouabain and veratridine observed when testing *Gambierdiscus* extracts could indicate the presence of MTXs. In this case if CTXs or substances that provoke CTX-like activity were present, CTX-like toxicity could not be detected. The second approximation is the evaluation of the cell viability in the presence of ouabain and veratridine. In this case, the presence of substances affecting voltage gated sodium channels will result in cell death, therefore, CTXs or other analogues having a similar mechanism of action will enhance cell mortality. With regard to the CTX standard for these assays, we are aware that many CTXs and precursors of CTXs have been described (Atlantic, India and Pacific) still, our reference molecule in this case is CTX1B and it was used for this purpose in all the experiments.

Our ANCOVA analysis and post-hoc Tukey’s H comparisons identified *G. excentricus* and *G. australes*, the two dominant species in the Canary Islands, [23], as producers of the highest levels of MTX like activity (Figure 4 and Table 1 and Table 2). The differences in the MTX-like response between these two species and *G. caribaeus, G. carolinianus* and *G. silvae* were significant (*p* < 0.05; Table 2).

High levels of MTX-like activity by *G. excentricus* strain VGO791 from Tenerife (Canary Islands) were reported by Fraga et al. [45] who estimate for this strain a value of 600 pg MTX eq cell^−1^. However, according to Pisapia et al. [46], the value estimated for the same strain, determined by ELA, was much lower (80 pg MTX eq cell^−1^). In addition to the different assay methods, the discrepancies in the results may have been due to the use of a crude extract [45] vs. aqueous methanol extracts after liquid-liquid partitioning [46]. In agreement with Fraga et al. [45], we measured very high levels of MTX - like activity by strain VGO791. The discrepancy with the results of Pisapia, et al. [46] suggest that liquid-liquid partitioning did not achieve a complete separation of MTXs and CTXs. Our evaluation of MTX-like response in 18 other strains of *G. excentricus* from the Canary Islands (Tenerife, La Palma and La Gomera) also showed that MTX-like activity was highest in this species, followed by *G. australes*.

High levels of MTX-like toxicity, similar to those found in *G. excentricus*, were also measured in 30 strains of *G. australes*, from Tenerife, Lanzarote, Gran Canaria, El Hierro, Fuerteventura and La Gomera (Figure 4). These results are in agreement with the MTX-like toxicity determined by ELA for a strain of *G. australes* isolated in Hawaii [48], but not for *G. australes* strains VGO1178, VGO1181 (both from Tenerife, Atlantic Ocean) and CCMP1653 (Hawaii, Pacific Ocean) Pisapia et al. [46]. For the latter three strains, MTX-like toxicity levels were low and the strains were less maitotoxic than the strains of the species *G. silvae, G. carolinianus* and *G. excentricus.* Strains VGO1178 and VGO1181 were also analysed in the present study and their MTX-like toxicities were much higher than determined by Pisapia et al. [46]. However, the analyses were conducted more than 2 years apart, which together with other (e.g., environmental, methodological) factors may explain the disparity of the results.

MTX-like response was also detected in the other three species of *Gambierdiscus* evaluated in this work, *G. caribaeus, G. silvae* and *G. carolinianus*. The differences between these three *Gambierdicus* species were not significant (*p* > 0.05; Table 2) and the levels were lower than those of *G. excentricus* and *G. australes.*

Literature reports suggest that MTX is of low oral toxicity and does not accumulate in fish flesh, but this conclusion is yet to be substantiated. Thus, whether MTX is able to induce CFP is unknown and need more investigation. A feeding study aimed at assessing MTX accumulation in the tissues of *Pagrus auratus* fed with *G. australes* showed the accumulation of MTX in the tissues of this carnivorous fish, including the viscera, liver and muscle [56]. Kohli et al. [56] reported that MTXs can accumulate in fish muscle. High MTX-type compounds levels were detected in microalgae from this study, especially in *G. excentricus* and *G. australes.* Therefore a considerable risk of presence of these very dangerous toxins in the flesh fish of *Seriola* spp. and *Epinephelus* spp. in the Canary Islands exists. Investigations of whether MTXs contribute to ciguatera outbreaks must therefore begin with determinations of the potential presence of these toxins in fish and their remnants, if available.

Among the species analysed in the present work, CTX-like response levels were highest in two strains of *G. excentricus* from La Gomera (VGO1361 and VGO1386, Figure 3). These results are in accordance with those reported for this species by other authors. For example, Litaker et al. [44] analysed eight *Gambierdiscus* species using a Neuro-2a assay and found that the most toxic was *G. excentricus*, specifically, a strain from Florida (USA). Also, in the above-described study by Fraga et al. very high levels of CTX-like activity were measured in a strain from Tenerife (VGO791). The high CTX-like response of *G. excentricus* is comparable to that of *G. polynesiensis*, the predominant CTX producer in the South Pacific [2,54].

In contrast to the consistent toxicity characteristics of *G. excentricus*, analyses of the toxicity of *G. australes* have yielded variable results. In our study, of the five analysed species, MTX activity was highest in *G. australes* and *G. excentricus*, but in three strains of *G. australes* CTX levels were ~ten-fold lower than those of the assayed *G. excentricus* strains. Pisapia et al. [46] also found a low range of CTX-like toxicity in three strains of *G. australes* differing in their origin (VGO1178 and VGO1181 from Canary Islands and CCMP1653 from Hawaii). Chinain et al. [2] reported comparatively low CTX-like toxicity for six *G. australes* strains originating from French Polynesia. Nishimura et al. [57] reported on a Japanese *G. australes* strain (S080911_1) with dichloromethane soluble fraction (DSF)—toxicity (Mousse bioassay, MBA) comparable to that of highly toxic *G. polynesiensis* species.

Only one strain of *G. caribaeus,* VGO1367, had a higher CTX-like toxicity than determined in other strains of the same species. This difference may reflect the different origin of the strains, as also observed in other species. For example, for two strains of *G. excentricus* from different sites (La Gomera Valle Rey-Ch. Condesa and La Gomera-San Sebastián), while high CTX-like toxicities characterised both, the respective values were markedly different. A comparison of those two strains with the published data of *G. excentricus* VGO791, from Tenerife Punta Hidalgo, also revealed considerable differences, as the latter strain has the highest toxicity detected thus far (1050 fg CTX1B cell^−1^) [45]. For strains of *G. australes* and *G. silvae*, however, the CTX-like response level was not remarkably related to their origin. For *G. carolinianus,* only one strain from the Canary Islands (VGO1197) was available for our study, such that its origin-dependent variability could not be evaluated.

In summary, our findings clearly show differences in the toxicities of different microalgal species and in the distribution of *Gambierdiscus* species between islands of the Canary Islands [22]. Moreover, the differing toxicities of different strains of the same species highlight the need to assess intraspecific and geographic variations in the distribution of CTX and MTX toxicity. This information will facilitate determinations of the risk of ciguatera in the Canary Islands. Nonetheless, other factors must also be taken into account, including the behaviour patterns of the two main fish species linked to ciguatera outbreaks in the Canary Islands (*Seriola* spp. and *Epinephelus* spp.), the fishing effort, age of the fish and the depth of the fishing platform.

The next essential step to improve the knowledge about Ciguatera intoxication is undoubtedly the determination of toxin profiles in *Gambierdiscus* species, mainly *G. excentricus* and *G. australes.* It is also necessary to compare the toxin profiles of fish captured in the same area. This information would be very useful to relate the content of CTXs produced by different Gambierdiscus species with their presence in fish captured in a particular area.

## 4. Conclusions

In this work, CTX-like and MTX-like total toxicities were evaluated in a neuro-2a assay and an ELA, respectively. This approach confirmed previous results of the high-level risk of CFP posed by strains of *G. excentricus*. Among the strains estimated quantitatively for CTX-like toxicity, the levels were highest in those of *G. excentricus* (ranging between 128.2 ± 25.68 and 510.6 ± 134.2 fg CTX1B eq cell^−1^), followed by *G. carolinianus* (100 ± 18.05 fg CTX1B cell^−1^).

The results of our study seem to demonstrate the possible presence of MTXs in *Gambierdiscus* species from the Canary Islands, mainly *G. australes*. A determination of the presence and concentration of MTXs in fish is needed in order to establish whether safety measures aimed at protecting human health should be implemented. Our characterisation of the toxicity *Gambierdiscus* strains is an important contribution to improving ciguatera risk assessments in the Canary Islands region.

## 5. Materials and Methods

### 5.1. Algal Cultures

Micro-algal culture isolates of *Gambierdiscus* spp. (*n* = 67) used in the analysis belong to the Culture Collection of Harmful Microalgae of the Spanish Institute of Oceanography (CCVIEO). Sixty-three strains were sourced from seven different locations in the Canary Islands. However, since there was only one strain of *G. carolinianus* from the Canary Islands, four additional strains from Cuba were added to the analysis, VGO1397, CUB-9B5, CUB-9C6 and CUB-9C8, all of them were analysed by ELA and the VGO1397 strain was also analysed by Neuro-2A.

The five species analysed were *G. australes* (*n* = 28), *G. excentricus* (*n* = 17), *G. caribaeus* (*n* = 10), *G. silvae* (*n* = 7) and *G. carolinianus* (*n* = 5). They were cultured in flasks containing different volumes of K/2 medium without silicates and prepared in seawater from the Ría de Vigo, with the salinity adjusted to 35. The cultures were incubated at 25 °C with a 12:12 L:D photoperiod.

Figure 1 shows all the species and strains from Canary Islands analysed as well as the type of analysis performed (ELA vs. Neuro-2a cell assay).

### 5.2. Neuro-2a Cell Assay

*Gambierdiscus* spp. cultures were cultivated until a biomass of at least 1.5 × 10^6^ cells was reached. Toxins were extracted following the method proposed by Caillaud et al. [3], with modifications. Briefly, the cells were harvested by filtration onto 47-mm Whatmann filters and, under sonication (2 min/extraction, Footswitch 40), were extracted twice with equal volumes of methanol (5 mL for 1 × 10^6^ cells) and twice with equal volumes of aqueous methanol (MeOH:H_2_O 50:50) followed by centrifugation at 10,395 × *g* for 10 min. The four supernatants were pooled and frozen at −20 °C until used. A volume of 1 mL of the pooled sample was filtered through 0.45-µm PTFE syringe filters and used for the Neuro-2a assay.

CTX-like toxicity was evaluated following the neuroblastoma cell-based (Neuro-2a) assay proposed by Caillaud et al. [49,58]. This assay measures cell viability of neuroblastoma cells (Neuro-2a) exposed to extracts when these extracts contain compounds that activate voltage gated sodium channels, that in the presence of ouabain and veratridine treatment cells die. Ouabain blocks the sodium and potassium ATPase pump inhibiting provoked efflux of sodium by compounds of the sample, and veratridine activates sodium compounds which increase and exacerbate the sodium levels.

Neuroblastoma murine cells (Neuro-2a) were obtained from the CIC cell bank of the University of Granada and maintained in RPMI-1640 medium (Gibco, Life Technologies, Carlsbad, CA, USA) supplemented with 5% foetal bovine serum (Sigma-Aldrich, St. Louis, MO, USA), 1 mM sodium pyruvate, 2.5 mg L-glutamine mL^−1^, 150 units penicillin mL^−1^ and 50 μg streptomycin mL^−1^ (Gibco Life Technologies, Carlsbad, CA), at 37 °C in a 5% CO2 humidified atmosphere, as previously described in Cañete and Diogéne [59].

Prior to the experiments, neuroblastoma cells were cultured in a 96-well microplate at 1.7 × 10^5^ cells mL^−1^ and incubated for 24 h under the same conditions as described for cell maintenance. Thereafter, triplicate samples of the cells were exposed for 24 h to eight concentrations (1/2 serial dilutions) of CTX1B standard from the Pacific region (provided by R. Lewis [6]) or *Gambierdiscus* extracts (serial dilutions) with or without ouabain and veratridine (O/V: to a final concentration of 0.14 mM and 0.014 mM respectively). In order to calibrate the experiments, the mean and standard deviation of the IC_50_ were estimated. Assays were done in triplicate. CTX-like toxicity was determined by measuring the cell viability using the colorimetric [3-(4,5-dimethylthiazol-2-yl)-2,5-diphenyltetrazolium] MTT (Sigma-Aldrich) [60] assay described in Manger et al. (1993) [61]. Absorbance was read at 570 nm using an automated multi-well scanning spectrophotometer (Biotek, Synergy HT, Winooski, VT, USA) and the results were expressed as a percentage of viability compared to the corresponding control (with and without O/V). The cell viability data were analysed using the statistical and programming software R 2.1.12 (R Foundation for Statistical Computing, Vienna, Austria, 2013) [62]. A dose–response curve fit with a sigmoid regression curve (with variable slope) was established for each experiment to estimate the concentration of *Gambierdiscus* extracts or standards that inhibited cell viability by 20% (IC_20_) and 80% (IC_80_) for each experimental condition (with and without O/V). Viabilities close to 50% (IC_50_) were further used as a toxicological parameter for qualitative and quantitative estimations of the content of CTX compounds produced, expressed as fg CTX1B equivalents (eq) per cell. The limit of detection was defined as the concentration inhibiting viability by 20% (IC_20_). Matrix effects of extracts were evaluated in wells lacking O/V. The CTX1B standard curve for this assay ranged from 25 to 0.2 pg·mL^−1^.

### 5.3. Erythrocyte Lysis Assay (ELA)

*Gambierdiscus* strains from Canarias listed in Figure 1 and strains from Cuba detailed in 5.1 section were analysed by ELA. Aliquots of 3 mL and 10 mL were removed from each culture for cell counting and MTX extraction respectively. The samples to be counted were fixed in Lugol solution and counted in a Sedgewick Rafter cell chamber. MTX was extracted by centrifuging the 10-mL aliquots at 2600 × g for 10 min at 4 °C. The cell-free supernatants were carefully discarded and 1200 µL of ELA buffer was added to the pelleted cells. These suspensions were transferred to 2-mL conical microcentrifuge tubes fitted with screw caps (Thermo Scientific, Waltham, MA, USA) and containing 0.5-mm glass beads (Soda Lime, Bio Spec Products, Inc., Bartlesville, OK, USA). Vigorous shaking of the samples for 20 s using a bead beater disrupted the cell membranes and released the cytoplasmic contents into the solution. Light microscopy observation was used to confirm that all cells were broken. The samples were maintained for 48 h at 4 °C until used in the assay.

Sheep blood in Alsever solution for use in the ELA was kindly provided by I. Manzano (CZ Veterinaria S.A., Porriño, Spain). Erythrocytes were separated from the plasma by centrifugation (400× *g*, 10 °C, 10 min), washed twice with ELA buffer and then diluted in ELA buffer (1:99) at a final concentration of ~1.7 × 10^7^ red cells mL^−1^. The ELA was conducted using Riobó’s method [63], modified as described by Holland et al. [48].

Each assay was carried out in triplicate in a 96-microwell plate by mixing 150 mL of blood with 150 mL of extract diluted in ELA buffer. Haemolysis controls for 0 and 100% lysis were prepared by mixing 150 µL of erythrocyte suspension with 150 µL of ELA buffer or distilled water, respectively. The microplates were incubated for 24 h at 4 °C after which the whole plate was centrifuged for 10 min at 400× *g* at 4 °C. From each well, 180 µL of supernatant was transferred to a new plate and the absorbance at 405 nm was measured using a microplate reader (BioRad model 550, BioRad Laboratories, Hercules, CA, USA). Measurements from three replicates were averaged.

### 5.4. Statistical Analysis

An analysis of covariance (ANCOVA) was used to test the effect of *Gambierdiscus* species and cell abundance on the response variable (percent haemolysis). Post-hoc comparisons between *Gambierdiscus* species were carried out using Tukey’s HSD. Normality, equal variance and the linearity assumptions of ANCOVA were previously checked.

All statistical analyses and graphic representations were performed using the statistical and programming software R 2.1.12 [62], packages ‘multcomp’ and ‘marmap’, available through the CRAN repository (www.r-project.org).

## Figures and Tables

**Table 1:** Analysis of covariance (ANCOVA) results of the effect of *Gambierdiscus* species and cells on haemolysis (%). Significant differences are indicated with asterisks (*).

Data	Df	Sum Sq	Mean Sq	F value	*p*	
Cells	1	0.93	0.93	79.34	1.41 × 10^−12^	***
Species	4	1.08	0.27	22.95	1.53 × 10^−11^	***
Residuals	60	0.70	0.01			

**Table 2:** Post-hoc Tukey’s H comparisons of the haemolysis (%) induced by five species of *Gambierdiscus* (*G. australes, G. caribaeus, G. carolinianus, G. excentricus* and *G. silvae*). Significant differences are indicated with asterisks (*).

Species	Estimate	Std. error	t-value	*p*	
*G. caribaeus-G. australes*	−0.33	0.05	−6.2	<0.001	***
*G. carolinianus-G. australes*	−0.26	0.07	−3.9	0.0018	**
*G. excentricus-G. australes*	0.21	0.03	6.17	<0.001	***
*G. silvae-G. australes*	−0.37	0.07	−5.34	<0.001	***
*G. carolinianus-G. caribaeus*	0.06	0.06	1.08	0.80	
*G. excentricus-G. caribaeus*	0.54	0.06	9.18	<0.001	***
*G. silvae-G. caribaeus*	−0.04	0.06	−0.78	0.93	
*G.excentricus -G. carolinianus*	0.47	0.07	6.59	<0.001	***
*G. silvae-G. carolinianus*	−0.11	0.06	−1.70	0.42	
*G. silvae-G. excentricus*	−0.58	0.08	−7.69	<0.001	***
